# The NADPH Oxidase Nox4 and Aging in the Heart

**DOI:** 10.18632/aging.100261

**Published:** 2010-12-27

**Authors:** Tetsuro Ago, Shouji Matsushima, Junya Kuroda, Daniela Zablocki, Takanari Kitazono, Junichi Sadoshima

**Affiliations:** ^1^Department of Cell Biology and Molecular Medicine, University of Medicine and Dentistry of New Jersey, New Jersey Medical School, Newark, NJ, USA; ^2^Department of Medicine and Clinical Science, Graduate School of Medical Sciences, Kyushu University, Fukuoka, Japan

**Keywords:** aging, ROS, mitochondria, NADPH oxidase, Nox4

## Abstract

Oxidative stress in mitochondria is believed to promote aging. Although passive leakage of electron from the mitochondrial electron transport chain has been considered as a major source of oxidative stress in the heart and the cardiomyocytes therein, enzymes actively producing reactive oxygen species may also exist in mitochondria. We have shown recently that Nox4, a member of the NADPH oxidase family, is localized on intracellular membranes, primarily at mitochondria, in cardiomyocytes. Mitochondrial expression of Nox4 is upregulated by cardiac stress and aging in the heart, where Nox4 could become a major source of oxidative stress. This raises an intriguing possibility that Nox4 may play an important role in mediating aging of the heart. Here we discuss the potential involvement of Nox4 in mitochondrial oxidative stress and aging in the heart.

## INTRODUCTION

Oxidative stress is defined as an excessive accumulation of reactive oxygen species (ROS) beyond the capacity of antioxidants [1]. ROS usually emerge as superoxide (O_2_^−^), which is dismutated immediately to hydrogen peroxide (H_2_O_2_) by superoxide dismutase (SOD). H_2_O_2_ is further converted into water by catalase or several types of peroxidases, including glutathione peroxidases and peroxiredoxin (Prx) (thioredoxin peroxidases) [2]. However, this sequential conversion from O_2_^−^ to water is not 100% efficient. Residual O_2_^−^ acts directly as an oxidant or reacts immediately with NO to produce peroxynitrite (ONOO^−^), a very harmful ROS, in NO-producing cells, such as endothelial cells [3] and macrophages [4]. H_2_O_2_ is stable and diffusible, but it can also be converted to hydroxyradical (OH−), a potent oxidant, by the Fenton reaction (Fe^2+^ + H_2_O_2_ → Fe^3+^ + OH**·** + OH^−^) [5].

In the heart, nearly 90% of ROS in cardiomyocytes is produced by leakage of electrons from Complexes I [6] and III [7] of the mitochondrial electron transport chain (ETC) during oxidative phosphorylation, an important process that generates ATP [8]. Since electrons are by-products of energy production by the ETC, O_2_^−^ production through this mechanism does not appear to be a regulated process. Although increased oxidative stress during heart failure is also largely mediated by the leakage of electrons from the mitochondrial ETC, whether or not the leakage of electrons is really the predominant source of oxidative stress in the heart under stress conditions remains to be elucidated. Besides the leakage from the ETC, ROS can be also generated through ROS-producing enzymes [9]. In particular, the NADPH oxidase family proteins are unique enzymes which purposefully produce O_2_^−^ [1,10,11]. An emerging hypothesis is that Nox4, a member of the NADPH oxidase family, is localized in mitochondria and actively produces ROS under pathological conditions and during aging [12,13]. Here we discuss the role of Nox4 in mediating oxidative stress in mitochondria and during aging in the heart.

## Regulation of aging and lifespan by oxidative stress in mitochondria

Aerobic cells need antioxidants localized in mitochondria in order to overcome inevitable ROS pro-duction following energy generation. A functional decline in the antioxidants or increased production of ROS in mitochondria causes accumulation of ROS, thereby leading to mitochondrial dysfunction. Accum-ulation of oxidative stress in mitochondria is highly relevant to aging and the development of various aging-related common diseases, including cardiovascular diseases. This hypothesis is referred to as the *“free radical theory of aging”* [8]*.* However, the involvement of specific forms of ROS and each antioxidant and/or ROS-producing enzyme in the process of aging remains obscure. Recently, generation of mouse models in which the level of specific ROS and/or enzymes modifying the level of ROS is altered has provided us with valuable information regarding the role of ROS in mediating aging in mammalian hearts.

There are 3 forms of superoxide dismutase (SOD 1-3), enzymes dismutating O_2_^−^, in mammals. SOD2 is localized specifically in the mitochondrial matrix. Since SOD2 has manganese in its reactive center, it is referred to as MnSOD. Systemic ablation of MnSOD in mice is accompanied by dilated cardiomyopathy and neurodegeneration leading to early postnatal death [14]. Cardiomyocyte-specific deletion of MnSOD was sufficient to induce mitochondrial dysfunction and the development of dilated cardiomyopathy. In this model, mitochondrial O_2_^−^ content was increased by 40% while H_2_O_2_ was decreased by 70% [15]. Thus, this model would be useful for determining the role of O_2_^−^ in mediating mitochondrial dysfunction and aging in the heart. Although overexpression of MnSOD extends lifespan in *Saccharomyces cerevisiae* [16] and *Drosophila melanogaster*[17], neither overexpression nor heterozygous knockout of MnSOD affects lifespan in mice [18,19], suggesting that mitochondrial O_2_^−^may not directly affect lifespan in mammals. However, MnSOD overexpression slows the replicative growth rate in human cancer cells [20]*.* Overexpression of MnSOD also protects the heart from mitochondrial dysfunction and heart failure in a mouse model of diabetic cardiomyopathy [21]. Thus, it is possible that O_2_^−^ may accelerate the aging processes by stimulating mitochondrial dysfunction in some organs and cell types.

In contrast to the ambiguous effect of O_2_^−^ upon lifespan/aging in mammals, mitochondrial H_2_O_2_ appears more clearly correlated with lifespan. In transgenic mice overexpressing catalase in mitochondria, maximal lifespan was extended by 20%, and cardiac pathology and development of cataracts were significantly delayed [22]. Possible underlying mechanisms include direct beneficial effects of reduced oxidative stress in mitochondria, *i.e.*preservation of mitochondrial functions, and indirect effects upon cell signaling modulated by the redox status. Catalase is primarily localized in peroxisomes, and is present in mitochondria only at low concentrations. Thus, mitochondrially-localized peroxiredoxin (Prx3 and 5) may be more critical in H_2_O_2_ detoxification [2]. Although the relevance of Prx5, but not Prx3, to lifespan has been demonstrated in *Drosophila melanogaster* [23], a gain-of-function allele of peroxiredoxin (thioredoxin peroxidase, Tsa1p) reduces oxidative stress but stimulates premature aging in yeast [24]. Thus, further investigation is required to elucidate how H_2_O_2_ is regulated in mitochondria, as well as the role of this mechanism in aging in the heart.

## Nox4 is a major superoxide-producing enzyme localized in mitochondria in the cardiovascular system

The NADPH oxidases are membrane-spanning proteins with NAD(P)H and FAD binding domains in their C-terminal tails that produce O_2_^−^ by transferring an electron from NADPH (or NADH) to molecular oxygen [11]. The NADPH oxidase was thought to be a phagocyte-specific enzyme and to play a critical role in mediating bacterial killing by producing a burst of O_2_^−^ [10], until its family protein Nox1 was discovered in smooth muscle cells and colonic epithelium [25]. There are now 7 known proteins in the NADPH oxidase (Nox) family, *i.e.* Nox1, Nox2, Nox3, Nox4, Nox5, Duox1 and Duox2, which, with the exception of Nox2, were all identified in this decade [1,10,11]. The initial cloning paper reported that Nox4 was expressed highly in the kidney [26,27]. However, in contrast to other Nox proteins that are expressed only in specific tissues or cells, Nox4 is ubiquitously expressed at high levels, including in cardiovascular systems, such as endothelial cells [28], smooth muscle cells [29], and cardiomyocytes [12,30]. As we discuss below, Nox4 has unique characteristics compared to the other members of the Nox family. In particular, its localization on intracellular membranes, including mitochondria, makes it an important candidate for regulating mitochondrial oxi-dative stress during aging in the heart.

## Mitochondrial localization of Nox4 in cardiomyocytes

Although Nox2, a prototypical NADPH oxidase, is localized primarily on the plasma membrane, Nox4 appears to be localized on intracellular membranes. Although the intracellular localization of Nox4 remains controversial, Nox4 appears to be localized in mitochondria in mesangial cells [13] and cardiomyocytes [12], the nucleus [31] or endoplasmic reticulum [32] in vascular endothelial cells, and the plasma membrane, especially at focal adhesions, in vascular smooth muscle cells [33]. The subcellular localizations of Nox4 may be truly cell type-dependent. However, different results may also be due to the distinct specificities of Nox4 antibodies used for analyses.

In cardiomyocytes, Nox4 significantly affects the level of oxidative stress in mitochondria [12,34]. Nox4 has a mitochondrial localization signal-like motif in the N-terminal region, which potentially directs expression of Nox4 to mitochondria [12]. Furthermore, many redox-sensitive mitochondrial proteins, including aconitase and components of Complex I and the MPTP, are significantly more oxidized in the mouse heart overexpressing Nox4, and less in the cardiac-specific Nox4 KO heart [12,34]. Thus, Nox4 not only produces O_2_^−^, thereby directly contributing to oxidative stress in mitochondria, but also induces oxidative damage of mitochondrial proteins and causes leakage of O_2_^−^ from mitochondria, which triggers a response known as ROS-induced ROS release [35]. Using cardiac-specific Nox4 KO mice, we have shown recently that Nox4 is an important source of oxidative stress in mitochondria during cardiac hypertrophy and failure [34]. The results suggest that electron leakage may not be the sole source of oxidative stress in the heart and that Nox4 in mitochondria could be an active source under stress. Since expression of Nox4 is upregulated by cardiac stress, including pressure overload, heart failure and aging, upregulation of Nox4 may allow it to be a significant source of mitochondrial oxidative stress under stress conditions.

The NADPH oxidases receive electrons from either NADPH or NADH and transfer them to molecular oxygen. The proto-type NADPH oxidase, Nox2, which is localized at the plasma membrane, utilizes NADPH as an electron donor [10]. During phagocytosis, the pentose-cycle supplying NADPH in the cytosol is drastically activated in phagocytes. In contrast, Nox4 appears to utilize NADH as an electron donor to produce O_2_^−^, at least in vitro assays [27,36,37]. Consistently, NADH produces a greater amount of ROS than NADPH in mitochondria isolated from hearts overexpressing Nox4 [12]. Since NADH is produced abundantly in the TCA cycle, Nox4 localized in mitochondria may directly utilize NADH derived from the TCA cycle to produce O_2_^−^. Thus, an exciting hypothesis is that Nox4 is an integral component of the mitochondrial TCA cycle and directly regulates the activity of NADH-generating enzymes as a negative feedback mechanism. When the TCA cycle operates, the resultant generation of NADH may increase ROS production through Nox4, which could in turn suppress the activity of mitochondrial proteins through oxidative modification.

## Aging, lifespan, and Nox4

The localization of Nox4 in mitochondria and its upregulation during aging support the hypothesis that Nox4 plays an important role in mediating ROS production during aging and controlling the aging process in the heart. Overexpression of Nox4 induces cellular senescence in fibroblasts [26,27] and apoptosis in cardiomyocytes [12]. In vascular smooth muscle cells, Nox4 upregulation plays a causal role in mediating the accumulation of polyploid cells, a biomarker of aging [38]. Cardiac-specific overexpression of Nox4 in mice exacerbates aging-associated cardiac pheno-types, such as left ventricular dysfunction, apoptosis, and fibrosis, accompanied by mitochondrial oxidative stress and dysfunction [12].

If endogenous Nox4 is a major source of oxidative stress and mediates aging in the heart, it could become an ideal target for pharmacological interventions to prevent age-associated complications in the heart. It will, therefore, be important to evaluate the role of endogenous Nox4 and Nox4-derived ROS in mediating aging of the heart, using Nox4 KO mice. We expect that downregulation of Nox4 should reduce the amount of O_2_^−^ in mitochondria, thereby inhibiting the aging process in the heart. On the other hand, since Nox4 consumes NADH as an electron donor to produce O_2_^−^, downregulation of Nox4 could lead to accumulation of NADH, a condition opposite from that induced by caloric restriction, an established intervention attenuating aging. Thus, the overall effect of Nox4 downregulation upon aging could be more complex. Although Nox4 does not have an obvious SOD-like motif in its structure, recent evidence suggests that Nox4 may directly produce H_2_O_2_ rather than O_2_^−^ [39,40]. If this observation is true, Nox4 may affect aging through production of H_2_O_2_. The involvement of specific ROS and the molecular mechanism mediating stimulation of aging by Nox4 in the heart remain to be elucidated. It should be noted that the mouse heart exhibits a remarkably negligible aging phenotype in response to Nox4 overexpression in young mice [12]. Increased expression of Nox4 in the heart upregulates antioxidants, such as catalase [12], indicating that the heart has adaptive mechanisms antagonizing the action of Nox4. Elucidating these mechanisms would also provide us with valuable information regarding how to retard aging in the heart.

## Effects of redox modification & Nox4 may be cell-type dependent

The effects of Nox4-derived ROS appear to be cell type-dependent. Nox4 stimulates apoptosis in endothelial cells [41] and cardiomyocytes [12], while it induces cell proliferation in smooth muscle cells [42] and cardiac fibroblasts [12]. Although the molecular mechanisms through which Nox4 exerts different effects in different cell types are currently unknown, it is possible that ROS generated by Nox4 affect distinct targets in each cell type. Interestingly, the response of the heart to pressure overload differs substantially between “cardiomyocyte-specific” [34] and “global” [30] Nox4 KO mice. Although cardiomyocytes are the major cell type in the heart, the heart also consists of other cell types, including cardiac fibroblasts, endothelial cells, and smooth muscle cells. The absence of Nox4 differentially affects each cell type and, thus, the overall response of the heart differs when Nox4 is deleted only in cardiomyocytes from when it is deleted in every cell type. By inference, the role of Nox4 in regulating aging could differ from tissue to tissue, and the role of Nox4 in regulating lifespan in the whole animal is probably complex. In this regard, the role of endogenous Nox4 in mediating aging in each organ should be addressed with both tissue-specific and systemic Nox4 KO mice.

## CONCLUSION

Unlike other ROS-producing enzymes, the Nox family proteins produce O_2_^−^ and/or H_2_O_2_ purposefully in a regulated manner. Nox4 is localized in the peri-nuclear region, especially in mitochondria, in cardiomyocytes. Due to its close proximity to mitochondrial proteins, ROS generated by Nox4 oxidize mitochondrial proteins, which in turn trigger mitochondrial dysfunction and electron leakage. We speculate that when ROS production via Nox4 is beyond the capacity of antioxidants, ROS are accumulated in mitochondria, thereby triggering the aging process. Aging not only upregulates Nox4 but also downregulates antioxidant mechanisms in mitochondria. We speculate that, as in the failing heart, Nox4 could be an important source of mitochondrial oxidative stress in the aging heart. If Nox4 is shown to be involved in the aging process in the heart, it could be a promising target of pharmacological intervention because aging-induced cardiomyopathy remarkably enhances the patient's risk of developing heart failure in response to many cardiac conditions, including high blood pressure, ischemia, and diabetes.
